# Intralymphatic immunotherapy with tyrosine-adsorbed allergens: a double-blind, placebo-controlled trial

**DOI:** 10.1186/s12931-021-01766-0

**Published:** 2021-06-04

**Authors:** Hye Jung Park, Sae-Hoon Kim, Yoo Seob Shin, Chul Hwan Park, Eun-Suk Cho, Seung Joon Choi, So Hyun Park, Joo Hyun Jung, Il Gyu Kang, Myoung Seok Lee, Dae Woo Kim, Sang Min Lee, Min-Suk Yang, Sang Pyo Lee

**Affiliations:** 1 Department of Internal Medicine, Gangnam Severance Hospital, Yonsei University College of Medicine, Seoul, Republic of Korea; 2 Division of Allergy and Clinical Immunology, Department of Internal Medicine, Seoul National University Bundang Hospital, Seoul National University College of Medicine, Seongnam, Republic of Korea; 3 Department of Allergy and Clinical Immunology, Ajou University Hospital, Ajou University College of Medicine, Suwon, Republic of Korea; 4 Department of Radiology and the Research Institute of Radiological Science, Gangnam Severance Hospital, Yonsei University College of Medicine, Seoul, Republic of Korea; 5 Department of Radiology, Gil Medical Center, Gachon University College of Medicine, Incheon, Republic of Korea; 6 Department of Otolaryngology-Head and Neck Surgery, Gil Medical Center, Gachon University College of Medicine, Incheon, Republic of Korea; 7 ENT-Over-Flower Clinic, Incheon, Republic of Korea; 8 Department of Radiology, SMG-SNU Boramae Medical Center, Seoul, Republic of Korea; 9 Department of Otorhinolaryngology Head and Neck Surgery, SMG-SNU Boramae Medical Center, Seoul, Republic of Korea; 10 Division of Pulmonology and Allergy, Department of Internal Medicine, Gil Medical Center, Gachon University College of Medicine, 21, Namdong-daero 774 beon-gil, Namdong-gu, Incheon, 21565 Republic of Korea; 11 Division of Allergy and Clinical Immunology, Department of Internal Medicine, SMG-SNU Boramae Medical Center, 20 Boramae-ro 5-gil, Dongjak-gu, Seoul, 07061 Republic of Korea

**Keywords:** Allergic rhinitis, Allergen immunotherapy, Intralymphatic injection, Treatment efficacy, Adverse events

## Abstract

**Background:**

Most previous studies used aluminum hydroxide-absorbed allergen extracts in evaluating the potential therapeutic roles of intralymphatic allergen-specific immunotherapy (ILAIT). In this study, we evaluated the therapeutic efficacy and safety of ILAIT with L-tyrosine-adsorbed allergen extracts of *Dermatophagoides farinae*, *D. pteronyssinus*, cat, dog, or mixtures thereof, in patients with allergic rhinitis induced by these allergens.

**Methods:**

In this randomized, double-blind, placebo-controlled trial, study subjects received three intralymphatic injections of L-tyrosine-adsorbed allergen extracts (active group) or saline (placebo group) at 4-week intervals.

**Results:**

Although ILAIT reduced daily medication use and skin reactivity to HDM and cat allergens at 4 months after treatment, overall symptom score on a visual analog scale (VAS), sinonasal outcome test-20 (SNOT-20), rhinoconjunctivitis quality of life questionnaire (RQLQ), daily symptom score (dSS), daily medication score (dMS), daily symptom medication score (dSMS), nasal reactivity to HDM allergen, and basophil activity to HDM, cat, and dog allergens at 4 months and 1 year after treatment were similar between the treatment and control groups. Intralymphatic injection was more painful than a venous puncture, and pain at the injection site was the most frequent local adverse event (12.8%); dyspnea and wheezing were the most common systemic adverse events (5.3%).

**Conclusions:**

ILAIT with L-tyrosine-adsorbed allergen extracts does not exhibit profound therapeutic efficacy in allergic rhinitis and can provoke moderate-to-severe systemic reactions and cause pain at the injection site.

*Trial registration:* clinicaltrials.gov: NCT02665754; date of registration: 28 January 2016

**Supplementary Information:**

The online version contains supplementary material available at 10.1186/s12931-021-01766-0.

## Background

Intralymphatic allergen-specific immunotherapy (ILAIT) was developed over a decade ago to overcome the shortcomings of conventional allergen-specific immunotherapy (AIT), such as systemic hypersensitivity reactions and the need for prolonged treatment [1]. The therapeutic effects of ILAIT have been demonstrated in numerous studies. Notably, three intralymphatic injections of allergen can alleviate pollen-induced allergic rhinitis (AR) [1–10], although one study reported no therapeutic effect after three or six intralymphatic injections of grass pollen allergen [11]. A few studies have assessed the efficacy and safety of ILAIT in patients with AR induced by inhalant allergens from cats, dogs, and house dust mites (HDM), such as *Dermatophagoides farinae* and *D. pteronyssinus* [12–16]. In ILAIT, aluminum hydroxide-absorbed allergen extracts were used in most studies [1–8, 10–13, 16], while aqueous allergen extracts were used in only one clinical trial [14, 15].

ILAIT has been shown to provoke mild local and systemic adverse reactions [1–4, 6, 8, 9, 11, 12, 16], although a few studies reported moderate-to-severe hypersensitivity reactions requiring intramuscular epinephrine injection or bronchodilator inhalation [5, 7, 14]. Thus, the clinical usefulness and safety of ILAIT using different allergen preparations remain unclear.

In this study, we evaluated the treatment outcomes and adverse effects of ILAIT using L-tyrosine-adsorbed allergen extracts of *D. farinae*, *D. pteronyssinus*, dog, cat, or mixtures thereof in patients with AR induced by one or more of these allergens.

## Methods

### Study subjects

The study was conducted at the outpatient clinics of five university hospitals in the Republic of Korea. From July 2016 to December 2018, the study included individuals with AR induced by individual or combined allergens from *D. farinae*, *D. pteronyssinus*, dogs, and cats. Patient selection was based on the following inclusion criteria [14, 15]. (1) Sensitization confirmed by a skin prick test (SPT) or allergen-specific immunoglobulin E (IgE) serum levels. Subjects with an allergen-to-histamine ratio of ≥ 1 or an allergen-specific IgE serum level of ≥ 0.35 kU/L were regarded as sensitized to an allergen. (2) Complaints of AR symptoms upon exposure to house dust, dogs, or cats. Patients with mild asthma and those sensitized to pollen allergens were included in the study if there was no definite allergic symptom during the pollen season. Patients with severe or uncontrolled asthma (forced expiratory volume in 1 s ≤ 50%, predicted); tyrosinemia; alkaptonuria; severe underlying conditions, including hepatic, renal, hematologic, oncologic, immunologic, infectious, or cardiovascular diseases; acute (within 1 month) or chronic respiratory diseases other than asthma, such as the common cold, flu, bronchiectasis, or chronic obstructive pulmonary disease; or previous AIT, were excluded. Pregnant or lactating women were also excluded. Written informed consent was obtained from all subjects included in the study.

### Randomization and blinding

Radiologists who performed intralymphatic injections randomly assigned subjects (1:1) into treatment and control groups using Microsoft Excel (Microsoft, Redmond, WA, USA) with a block size of 10. Other researchers prescribing medication and assessing treatment outcomes and adverse events were blinded to the treatment; blinding was maintained until all assessments and recordings were completed.

### Intervention

Subjects in the active group were administered three injections (0.1 mL) of the respective allergen extract at 4-week intervals. Using ultrasound guidance and a 25-gauge needle, L-tyrosine-adsorbed allergen extracts (Tyrosine S®; Allergy Therapeutics, Worthing, UK) were aseptically injected into the superficial inguinal lymph node in the right side of the groin. Before the injections, aspiration was performed to avoid inadvertent intravascular administration. After each injection, subjects were closely monitored for 1 h, and vital signs were assessed at 5-min intervals; any adverse events were recorded. Adverse events due to previous injections were also evaluated before the next injection. Allergens were initially administered at a 1000-fold dilution of the highest dose used for subcutaneous immunotherapy (SCIT) [14]. For the second and third injection, the administered dose was 3 and 10 times the initial allergen concentration, respectively [6, 12, 14]. Subjects in the control group received three injections (0.1 mL) of normal saline at 4-week intervals.

### Outcome measures

**Fig. 1:**
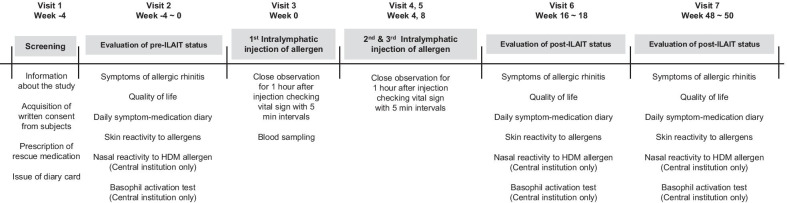
Study outline. At visit 1, rescue medications were prescribed, and diary cards were issued after informed consents were obtained; at visit 2, pre-ILAIT status was evaluated; at visits 3 to 5, subjects received intralymphatic immunotherapeutic allergen injection; at visits 6 to 7, post-ILAIT status was evaluated, respectively. *ILAIT* intralymphatic allergen-specific immunotherapy, *HDM* house dust mite

Patients were asked to answer the questionnaires and complete diary that measured allergic symptoms and rescue medication, as well as underwent a SPT and intradermal test (IDT) before treatment, at 4 months after treatment, and at 1 year after treatment (Fig. 1) [14]. For subjects who were enrolled at the central institution and whose target allergens were HDMs, a nasal allergen provocation test (NAPT) was also performed [14].

#### Overall treatment effects

Subjects were asked to score their overall AR symptoms on a visual analog scale (VAS) ranging from 0 (none) to 100 (extremely severe).

#### QOL

Quality of life (QOL) was evaluated using the Korean version of the sinonasal outcome test-20 (SNOT-20) and rhinoconjunctivitis QOL questionnaire (RQLQ) [14, 17, 18]. The RQLQ score was used as the primary outcome measure.

#### Symptom medication score

Subjects self-reported a daily symptom score (dSS) for runny nose, blocked nose, sneezing, and itchy nose the month before treatment, as well as at 4 months and 1 year after treatment. For the dSS, a four-point scale was used: 0, no symptoms; (1) mild symptoms (easily tolerated); (2) moderate symptoms (bothersome but tolerable); (3) severe symptoms (hard to tolerate and interfering with daily activities) [19]. The patients were provided antihistamines or nasal glucocorticosteroid spray in a stepwise fashion according to Allergic Rhinitis and its Impact on Asthma (ARIA) recommendations [14, 19–21]. Rhinitis medication use was scored to obtain the daily medication score (dMS), and the daily symptom medication score (dSMS) was calculated as the sum of the dSS and dMS [19].

#### SPT and IDT

The SPT and IDT were performed using serially diluted extracts of the respective allergen (*D. farinae*, *D. pteronyssinus*, dog, or cat; Tyrosine S®; Allergy Therapeutics). The skin test results were interpreted after 15 min by measuring the mean wheal diameter induced by each allergen, using calipers with resolution of 0.01 mm.

#### NAPT

Subjects sensitized to HDMs underwent NAPT at the central institution with saline and freshly reconstituted freeze-dried allergen solutions of Der f 1 (0.04, 0.4, 1, 2, and 4 μg/mL) at 15-min intervals [14, 22]. At each step, two puffs (0.05 mL each) of the solution at room temperature were applied to each nasal passage using a metered pump spray; nasal obstruction, rhinorrhea, itching, sneezing, and ocular symptoms were recorded by placing a vertical mark on a horizontal 100 mm line or using a VAS, with the total VAS score calculated as the sum of five VAS scores (total range, 0–500) [14, 22, 23]. Measurements were taken before the NAPT (baseline VAS) and at 15 min after each challenge [14]. Acoustic rhinometry was performed using an SRE 2000 rhinometer (Rhinometrics, Lynge, Denmark) according to the guidelines of the Standardization Committee on Acoustic Rhinometry [24]. The mean volume (cm^3^) in the anterior nasal segment (2–6-cm volume) was measured before the NAPT (baseline test) and at 15 min after each challenge [14, 22]. Following the protocol in a previous report [4], the total VAS score and the 2–6 cm volume at different time points (15 min intervals) were plotted. The area under the curve (AUC) values for those two variables were calculated for every patient.

#### Basophil activation test

Blood was drawn (at the central institution) from 15 subjects (eight in the active group and seven in the control group) for a basophil activation test (BAT) before treatment, as well as at 4 months and 1 year after treatment. The cells were stimulated with the respective allergen within 2 h of blood sampling using a commercially available Flow-CAST kit (Bühlmann Laboratories AG, Schönenbuch, Switzerland) according to the manufacturer’s and previously described instructions [25].

### Statistical analyses

Statistical analyses were performed using PASW 20.0 (SPSS Inc. Chicago, IL, USA) and GraphPad Prism 6.01 software (GraphPad Software, La Jolla, CA, USA). Continuous variables were analyzed with the Man-Whitney *U* test for intergroup analysis, and the Wilcoxon signed-rank test for intragroup analysis. Categorical variables were analyzed with the χ^2^ test or Fisher’s exact test. *P-*values < 0.05 were considered to indicate statistical significance.

### Minimum sample size calculation

A power calculation was performed with a two-sample *t*-test for the primary outcome (mean total score of RQLQ). We expected a clinically significant score difference of 0.50 in mean total score of RQLQ between the two groups at 4 months after treatment [26]. Assuming a standard deviation of 0.61 based on a previous pilot study [14], and aiming for a power of 0.80, type-1 error rate of 0.05, and loss to follow-up rate of 40%, the required total sample size was calculated to be 38.

### Ethics

The study was conducted in accordance with good clinical practice guidelines [27]. The study was approved by the Ministry of Food and Drug Safety of the Republic of Korea (20,160,004,728) and our institutional review boards (GBIRB2016-002, 3-2017-0307, 26-2017-45, B1707-409-401, and AJIRB-MED-CT1-17-098). The study was monitored by our human research protection committees and registered in an open-access trial registry (ClinicalTrials.gov identifier: NCT02665754).

## Results

### Study subjects

**Fig. 2:**
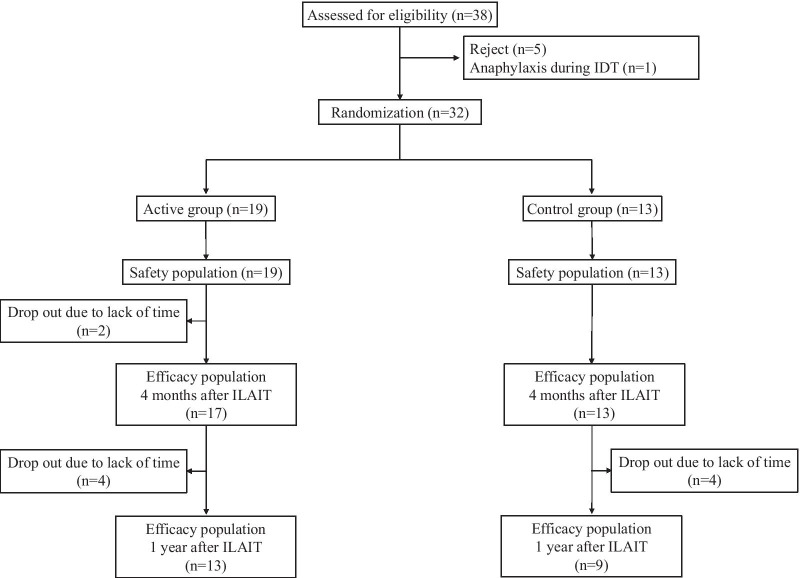
Participant flowchart. *IDT* intradermal test, *ILAIT* intralymphatic allergen-specific immunotherapy

A schematic representation of patient selection is shown in Fig. 2. Thirty-eight subjects were initially enrolled in the study; among them, five subjects rejected to be enrolled, and one developed an anaphylactic reaction during the IDT with a target allergen. Thirty-two subjects were randomly allocated into treatment (n = 19) and control (n = 13) groups, and each subject received three intralymphatic injections of target allergens or a placebo; one patient in the active group withdrew from the study after receiving only one injection but was still considered for the ILAIT safety evaluation. Another subject in the active group withdrew from the study after receiving three intralymphatic injections due to a lack of time. The therapeutic efficacy of ILAIT was evaluated (in 17 subjects in the active group and 13 in the control group) at 4 months after the first intralymphatic injection. Subsequently, the therapeutic efficacy of ILAIT was re-accessed at 1 year after the first intralymphatic injection; as eight subjects (four in the active group and four in the placebo group) withdrew due to lack of time, the 1-year follow-up was conducted for 22 subjects (13 in the active group and nine in the control group).

The baseline characteristics of the study subjects are summarized in Table 1. The mean age was 34.2 ± 9.5 years, and 19 subjects (59.4%) were female. The mean symptom duration of AR was 14.5 ± 9.5 years. Subjects in the active group were younger than those in the control group (32.4 ± 11.1 years vs. 36.9 ± 5.9 years, *P* = 0.037). By contrast, gender and symptom duration did not differ significantly between the two groups. The most common allergic comorbidity was asthma (59.4%), followed by urticaria (21.9%), food allergy (18.8%), drug allergy (15.6%), and atopic dermatitis (12.5%). Twenty-one subjects (65.6%) had a family history of allergy. The frequencies of comorbid allergic diseases and a family history of allergy did not differ significantly between the two groups. The target allergens were *D. farinae*, *D. pteronyssinus*, cat, and dog extracts for 22 (68.8%), 21 (65.6%), 19 (59.4%), and 8 (25.0%) subjects, respectively; no significant differences in the frequencies of each target allergen were observed between the groups. Among the subjects followed-up at 4 months and 1 year, age, gender, symptom duration, allergic comorbidities, family history of allergy, and target allergens did not differ between the two groups.

**Table 1:** Baseline characteristics of the study subjects

	Cohort for testing procedural safety				Cohort for testing treatment efficacy (at 4 months after ILAIT)				Cohort for testing treatment efficacy (at 1 year after ILAIT)			
	All subjects(*n* = 32)	Active group (*n* = 19)	Control group (*n* = 13)	*P*-value	All subjects (*n* = 30)	Active group (*n* = 17)	Control group (*n* = 13)	*P*-value	All subjects (*n* = 22)	Active group (*n* = 13)	Control group (*n* = 9)	*P*-value
Age, year	34.2 ± 9.5	32.4 ± 11.1	36.9 ± 5.9	**0.037**	34.9 ± 9.3	33.4 ± 11.2	36.9 ± 5.9	0.079	36.5 ± 9.7	35.2 ± 11.8	38.4 ± 5.7	0.186
No. of Female	19 (59.4)	11 (57.9)	8 (61.5)	0.565	18 (60.0)	9 (52.9)	8 (61.5)	0.410	13 (59.1)	7 (53.8)	6 (66.7)	0.439
Symptom duration, years	14.5 ± 9.5	15.8 ± 8.7	12.8 ± 10.6	0.648	14.2 ± 9.6	15.4 ± 9.0	12.8 ± 10.6	0.674	14.5 ± 10.0	13.6 ± 10.3	15.4 ± 10.3	0.645
Comorbid allergic diseases												
Asthma	19 (59.4)	11 (57.9)	8 (61.5)	0.470	18 (60.0)	10 (58.8)	8 (61.5)	0.508	13 (59.0%)	7 (53.8)	6 (66.7)	0.436
Urticaria	7 (21.9)	6 (31.6)	1 (7.7)	0.186	6 (20.0)	5 (29.4)	1 (7.7)	0.240	4 (18.2)	4 (30.8)	0 (0.0)	0.184
Food allergy	6 (18.8)	5 (26.3)	1 (7.7)	0.294	6 (20.0)	5 (29.4)	1 (7.7)	0.240	5 (22.7)	4 (30.8)	1 (11.1)	0.423
Drug allergy	5 (15.6)	3 (15.8)	2 (15.4)	0.660	5 (16.6)	3 (17.7)	2 (15.4)	0.657	4 (18.1)	3 (23.1)	1 (11.1)	0.652
Atopic dermatitis	4 (12.5)	2 (10.5)	2 (15.4)	0.542	4 (13.3)	2 (11.8)	2 (15.4)	0.591	3 (13.6)	2 (15.4)	1 (11.1)	0.642
Family history of allergic diseases	21 (65.6)	11 (57.9)	10 (76.9)	0.283	20 (66.7)	10 (58.8)	10 (76.9)	0.259	15 (68.2)	8 (61.5)	7 (77.8)	0.372
Target allergen												
*Dermatophagoides farinae*	22 (68.8)	13 (68.4)	9 (69.2)	0.636	20 (66.7)	11 (64.7)	9 (69.2)	0.554	15 (68.2)	8 (61.5)	7 (77.8)	0.372
*D. pteronyssinus*	21 (65.6)	13 (68.4)	8 (61.5)	0.487	19 (63.3)	11 (64.7)	8 (61.5)	0.579	14 (63.6)	8 (61.5)	6 (66.7)	0.584
Cat	19 (59.4)	10 (52.6)	9 (69.2)	0.285	18 (60.0)	9 (52.9)	9 (69.2)	0.301	12 (54.5)	7 (53.8)	5 (55.6)	0.639
Dog	8 (25.0)	5 (26.3)	3 (23.1)	0.587	7 (23.3)	4 (23.5)	3 (23.1)	0.660	6 (27.3)	3 (23.1)	3 (33.3)	0.477

Data are shown as the means ± standard deviations (SDs) or frequencies (%). *ILAIT* intralymphatic allergen-specific immunotherapy

### Safety of ILAIT

**Fig. 3:**
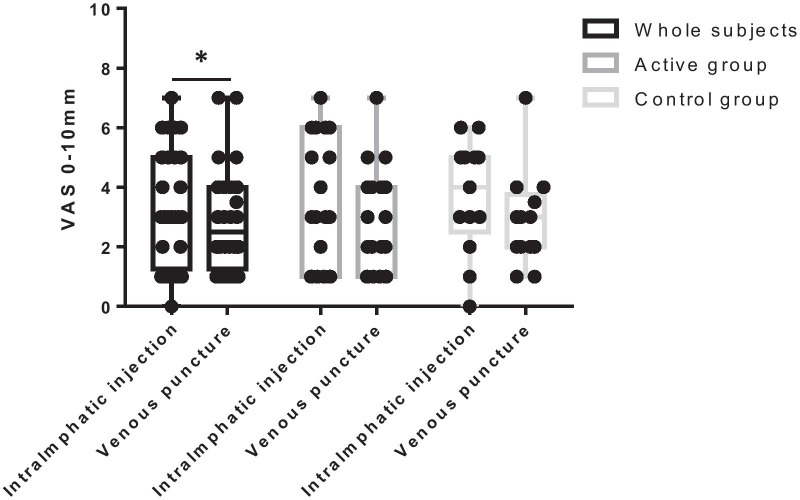
Pain of intralymphatic injection. On the day of the first intralymphatic injection, blood sampling was also performed. subjects were asked to compare the pain of intralymphatic injection to that of a venous puncture. Box plots show median (line), 24th and 75 percentiles (box), and ranges (whiskers). *P < 0.05

On the day of the first intralymphatic injection, subjects reported that intralymphatic injection-caused pain was mild but more intense than venous punctures (VAS score: 3.5 ± 2.0 mm vs. 2.8 ± 1.7 mm, *P* = 0.019; Fig. 3). Although intralymphatic injection seemed to be more painful than venous punctures, the difference in pain was not significant within each group (treatment-group VAS scores: 3.4 ± 2.1 mm vs. 2.8 ± 1.8 mm, *P* = 0.092; control VAS scores: 3.7 ± 1.9 mm vs. 2.9 ± 1.6 mm, *P* = 0.106). The extent of pain caused by intralymphatic injection and venous puncture did not differ significantly between the two groups.

Local and systemic adverse events are summarized in Table 2. Pain was the most frequent local adverse event (12.8%), followed by itchiness (8.5%), paresthesia (6.4%), wheal (1.1%), and sensation of heat (1.1%) at the site of intralymphatic injection. The most frequent systemic adverse events were dyspnea (5.3%) and wheezing (5.3%), followed by chest discomfort (4.3%), headache (3.2%), chills (3.2%), palpitation (2.1%), urticaria (2.1%), itchy eyes (2.1%), itchy palms (2.1%), abdominal discomfort (1.1%), fever (1.1%), rhinorrhea (1.1%), postnasal drip (1.1%), and sneezing (1.1%). No significant differences in systemic and local adverse events were observed between the two groups. According to Müller’s classification, four (one in the active group and three in the control group) and one (in the active group) subjects developed systemic hypersensitivity reactions of grade 1 and grade 3, respectively. The subject that developed a severe hypersensitivity reaction (grade 3) after the first intralymphatic injection complained of paresthesia at the injection site accompanied by dyspnea, chest and abdominal discomfort, palpitation, chills, and itchy palms. Although the allergen was further diluted 1,000-fold for the second intralymphatic injection, the subject experienced mild paresthesia at the injection site. The allergen’s concentration was increased tenfold for the third intralymphatic injection, and the subject complained of pain and paresthesia at the injection site accompanied by fever and chills. For other subjects in the active group who had no reactions or a mild hypersensitivity reaction (grade 0–1), allergen concentrations were increased three-fold for the second intralymphatic injection and tenfold for the third intralymphatic injection.

**Table 2:** Adverse events associated with the intralymphatic injections in the cohort for testing procedural safety (n = 32)

	All subjects (94 injections)	Active group (55 injections)	Control group (39 injections)	*P*-value
Local reactions				
Pain	12 (12.8)	9 (16.4)	3 (7.7)	0.178
Itch	8 (8.5)	6 (10.9)	2 (5.1)	0.275
Paresthesia	6 (6.4)	4 (7.3)	2 (5.1)	0.513
Wheal	1 (1.1)	0 (0.0)	1 (1.8)	0.415
Heat sensation	1 (1.1)	1 (1.8)	0 (0.0)	0.585
Systemic reaction				
Dyspnea	5 (5.3)	3 (5.5)	2 (5.1)	0.660
Wheezing	5 (5.3)	3 (5.6)	2 (5.1)	0.660
Chest discomfort	4 (4.3)	4 (7.3)	0 (0.0)	0.112
Headache	3 (3.2)	2 (3.6)	1 (2.6)	0.628
Chills	3 (3.2)	2 (3.6)	1 (2.6)	0.628
Palpitation	2 (2.1)	2 (3.6)	0 (0.0)	0.340
Urticaria	2 (2.1)	1 (1.8)	1 (2.6)	0.660
Itchy eyes	2 (2.1)	0 (0.0)	2 (5.1)	0.170
Itchy palms	1 (1.1)	1 (1.8)	0 (0.0)	0.585
Abdominal discomfort	1 (1.1)	1 (1.8)	0 (0.0)	0.585
Fever	1 (1.1)	1 (1.8)	0 (0.0)	0.585
Rhinorrhea	1 (1.1)	0 (0.0)	1 (2.6)	0.415
Postnasal drip	1 (1.1)	0 (0.0)	1 (2.6)	0.415
Sneezing	1 (1.1)	0 (0.0)	1 (2.6)	0.415
Hypersensitivity reactions*				0.273
Grade 0 (none)	88 (94.6)	52 (94.5)	36 (94.7)	
Grade 1 (mild)	4 (4.3)	1 (1.8)	3 (7.7)	
Grade 2 (moderate)	0 (0.0)	0 (0.0)	0 (0.0)	
Grade 3 (severe)	1 (1.1)	1 (1.8)	0 (0.0)	
Grade 4 (anaphylactic)	0 (0.0)	0 (0.0)	0 (0.0)	

Data are shown in frequencies (%). *Hypersensitivity reactions were graded according to Müller's classification

### Symptom relief and rescue medication use

**Fig. 4:**
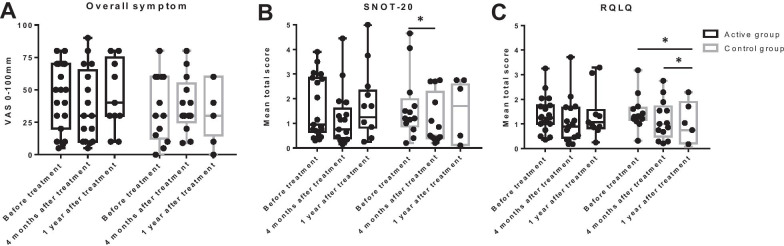
Symptom relief and quality of life. **A** Subject-reported overall symptom of allergic rhinitis. Study subjects were asked to score their overall symptom of allergic rhinitis on a VAS. **B** Total mean score of SNOT-20. **C** Total mean score of RQLQ. *P < 0.05. *VAS* visual analog scale, *SNOT-20* sinonasal outcome test-20, *RQLQ* rhinoconjunctivitis quality of life questionnaire

The overall AR symptoms scored on a VAS did not change significantly in the treatment or control groups at 4 months or 1 year after treatment, and the scores did not differ between active and control groups at 4 months (VAS score: 37.9 ± 27.6 mm vs. 38.4 ± 21.7 mm, *P* = 0.805) and 1 year (VAS score: 47.7 ± 26.5 mm vs. 33.3 ± 22.9 mm, *P* = 0.186) after treatment (Fig. 4A). In the active group, the total mean scores of SNOT-20 and RQLQ and the mean domain score of the emotional function domain in RQLQ did not change significantly at 4 months or 1 year after treatment; however, these scores were significantly reduced at 4 months and/or 1 year after treatment in the control group (Fig. 4B—C and Additional file 1:Fig. S1A). The mean total scores of SNOT-20 and RQLQ and the mean domain score of the emotional function domain in RQLQ did not differ between active and control groups at 4 months (SNOT-20: 1.12 ± 1.12 vs. 1.12 ± 1.01, *P* = 0.773; RQLQ: 1.10 ± 0.88 vs. 1.16 ± 0.79, *P* = 0.805; the emotional function domain in RQLQ: 1.03 ± 0.82 vs. 1.02 ± 1.04, *P* = 0.742) and 1 year (SNOT-20: 1.83 ± 1.63 vs. 1.43 ± 1.20, *P* = 0.695; RQLQ: 1.35 ± 0.89 vs. 1.01 ± 0.83, *P* = 0.262; the emotional function domain in RQLQ: 1.04 ± 0.89 vs. 0.92 ± 1.14, *P* = 0.471) after treatment. Mean domain scores of practical problem, sleep, and non-nose/eye symptoms domain in RQLQ decreased significantly at 4 months and/or 1 year after treatment in both groups (Additional file 1:Fig. S1B–D). They were not different between active and control groups at 4 months (the practical problem domain: 0.90 ± 0.90 vs. 1.04 ± 0.94, *P* = 0.711; the sleep domain: 0.94 ± 1.10 vs. 0.90 ± 0.69, *P* = 0.711; the non-nose/eye symptoms domain: 1.04 ± 1.18 vs. 1.14 ± 1.00, *P* = 0.621) and 1 year (the practical problem domain: 1.29 ± 1.14 vs. 0.97 ± 0.91, *P* = 0.601; the sleep domain: 1.51 ± 1.23 vs. 0.93 ± 0.88, *P* = 0.262; the non-nose/eye symptoms domain: 1.40 ± 1.19 vs. 1.04 ± 0.87, *P* = 0.556) after treatment. Meanwhile, mean domain scores of nasal and ocular symptoms domain in RQLQ did not change during the study period in either group, nor did they differ between active and control groups at 4 months (the nasal symptoms domain: 1.62 ± 0.90 vs. 1.50 ± 0.77, *P* = 0.773; the ocular symptoms domain: 1.19 ± 0.90 vs. 1.21 ± 1.12, *P* = 0.837) and 1 year (the nasal symptoms domain: 1.79 ± 0.86 vs. 1.33 ± 0.98, *P* = 0.324; the ocular symptoms domain: 1.02 ± 0.92 vs. 0.86 ± 0.87, *P* = 0.601) after treatment (Additional file 1: Fig. S1E, F).

**Fig. 5:**
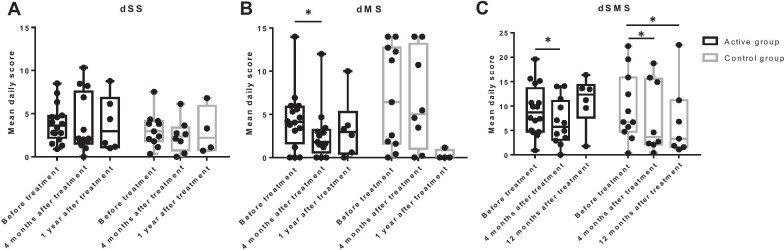
Daily symptom and medication use. **A** daily symptom score (dSS). **B** daily medication score (dMS). **C** daily symptom medication score (dSMS). *P < 0.05

Diaries of daily symptom and medication were returned by 27 subjects (16 in the active group and 11 in the control group); however, only 21 (13 in the active group and eight in the control group) and 10 (six in the active group and four in the control group) completed the reports for the 4-month and 1-year follow-up, respectively. According to these reports, the dSS did not change significantly in either group (Fig. 5A). The dSS did not differ between active and control groups at 4 months (mean daily score: 3.8 ± 3.4 vs. 2.4 ± 1.9, *P* = 0.750) and 1 year (mean daily score: 3.8 ± 3.2 vs. 3.0 ± 2.8, *P* = 0.610) after treatment. Meanwhile, the dMS decreased significantly at 4 months after treatment in the active group but not in the control group (Fig. 5B). However, the dMS was not different between active and control groups at 4 months (mean daily score: 2.7 ± 3.1 vs. 6.6 ± 5.7, *P* = 0.076) and 1 year (mean daily score: 3.4 ± 3.6 vs. 0.3 ± 0.6, *P* = 0.067) after treatment, which may be due to the small number of subjects who completed the diary. In both groups, the dSMS decreased significantly at 4 months and/or at 1 year after treatment (Fig. 5C). The dSMS was not different between active and control groups at 4 months (mean daily score: 6.7 ± 4.7 vs. 7.7 ± 7.4, *P* = 0.970) and 1 year (mean daily score: 11.0 ± 5.1 vs. 6.9 ± 7.8, *P* = 0.181) after treatment.

### Skin reactivity

In the active group, skin reactivity to serial dilutions of HDM allergen decreased at 4 months after treatment (*P* < 0.05 for the 10^3^-fold dilution), then this reduction in skin reactivity diminished at 1 year after treatment, however the reduction in skin reactivity to 10^3^- and tenfold dilutions of HDM allergen remained significant (Additional file 2: Fig. S2A). On the contrary, in control subjects, skin reactivity after exposure to serial dilutions of HDM allergen increased at 4 months after treatment (*P* < 0.05 for the 10^7^-, 10^6^-, 10^5^-, 10^4^-, 10^3^-, and 10^2^-fold dilutions compared with baseline).

Skin reactivity to serial dilutions of cat allergens decreased at 4 months after treatment in the active group, and it was significantly lower than that in the control group (*P* < 0.05 for the 10^7^-, 10^6^-, 10^5^-, 10^4^-, and 10^3^-fold dilutions compared with the control group), then this reduction in skin reactivity diminished at 1 year after treatment (Additional file 2: Fig. S2B). In control subjects, skin reactivity to serial dilutions of cat allergens generally increased at 4 months, then decreased at 1 year after treatment, but the changes were not significant.

In the active group, skin reactivity after exposure to serial dilutions of dog allergens decreased at 4 months after treatment and increased at 1 year after treatment (Additional file 2: Fig. S2C). In control subjects, skin reactivity to serially diluted dog allergens increased at 4 months and 1 year after treatment; nevertheless, skin reactivity changes were not statistically significant in either group.

The IDT results revealed that skin reactivity to serially diluted HDM, cat, and dog allergens did not change significantly during the study period and that no differences existed between the two groups (Additional file 3: Fig. S3).

### Nasal reactivity

**Fig. 6:**
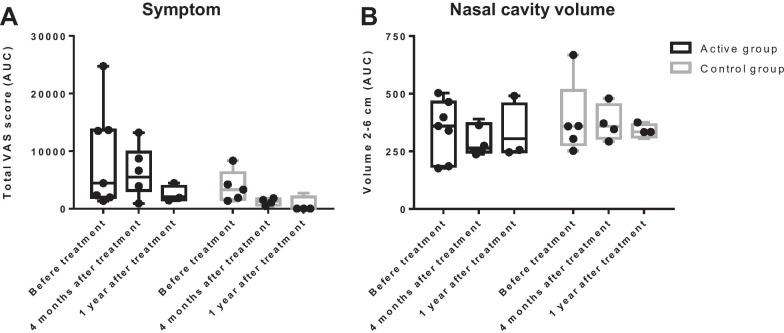
Nasal reactivity in NAPT with serially diluted *Dermatophagoides farinae* allergen. **A** The AUC of the sum of 5 VAS scores (total range 0–500). **B** The AUC of the mean value of volume (cm^3^) in the anterior nasal segment (Volume 2–6 cm). VAS, visual analogue scale; AUC, area under curve

Among the 12 subjects who underwent the NAPT with the *D. farinae* allergen, nasal and ocular symptoms decreased at 4 months and 1 year after treatment in both groups; however, these changes were not statistically significant (Fig. 6A). Similarly, no significant alterations in nasal cavity volume were observed during the study period (Fig. 6B). Nasal or ocular symptoms and nasal cavity volume decrease during NAPT were not different between two groups before treatment and at 4 months and 1 year after treatment.

### Basophil reactivity

Among the 15 subjects who provided blood samples, the percentages of activated CD63^+^ basophils after in vitro stimulation with *D. farinae*, *D. pteronyssinus*, and cat allergens, decreased at 4 months and 1 year after treatment in the active group, although not significantly. No changes in the percentages of activated CD63^+^ basophils were observed in control subjects (Additional file 4: Fig. S4A–C). We could not evaluate the changes in the percentage of activated CD63^+^ basophils after in vitro stimulation with dog allergens due to the small number of subjects (two in the active group and one in the control group; Additional file 4: Fig. S4D.

## Discussion

In 2008, Senti et al. first described ILAIT as a promising AIT that can alleviate nasal reactivity to pollen allergens as fast as within 4 months after treatment, which is significantly faster than with SCIT. In the open-label randomized study by Senti et al., ILAIT alleviated nasal reactivity for up to 3 years in patients with pollen-induced AR [1]. Moreover, ILAIT has been shown to reduce allergic symptoms, serum pollen-specific IgE levels, and skin reactivity to the allergen for up to 3 years after treatment. Notably, compared with patients treated with SCIT, fewer ILAIT-treated patients required rescue medications. Subsequent open-label pilot studies confirmed that ILAIT alleviated nasal or ocular symptoms, improved the QOL, decreased nasal reactivity as observed in the NAPT and allergen-specific IgE serum levels, as well as increased allergen-specific IgG4 serum levels and the number of plasmablasts producing grass pollen-specific immunoglobulins other than IgE [5, 7]. However, nasal reactivity, nasal and ocular symptoms, and QOL are largely self-reported and subject to confounding effects, thus introducing bias and uncertainty regarding the therapeutic efficacy of AIT. Therefore, double-blind placebo-controlled (DBPC) trials are required to elucidate the clinical efficacy of AIT objectively [28, 29].

Thus far, six DBPC trials have evaluated the therapeutic efficacy and adverse effects of ILAIT in patients with pollen-induced AR [2–4, 6, 8–11]. In a DBPC trial with 15 subjects, Hylander et al. reported that ILAIT with birch or grass pollen improved seasonal allergic symptoms, decreased rescue medication use, and increased the activation levels of CD4^+^ T cells in the peripheral blood [2]. ILAIT also alleviated nasal reactivity to allergens and reduced the levels of the proinflammatory cytokine interleukin (IL)-8 in nasal fluids, although these effects were not statistically significant.

In a follow-up study with 20 additional subjects, they found that ILAIT modestly alleviated seasonal allergic symptoms. Moreover, ILAIT decreased nasal reactivity and IgG4 affinity to allergens in individuals who exhibited symptom improvement, although nasal reactivity did not differ significantly between treated and control subjects [3]. In a subsequent clinical trial with 60 patients sensitized to both birch and grass pollen allergens, ILAIT significantly improved the QOL during birch pollen but not grass pollen season [4]. They also found that ILAIT decreased rescue medication use during birch and grass pollen seasons, as well as reduced nasal reactivity to grass pollen and skin reactivity to birch and grass pollen; however, the overall improvement in symptoms did not differ significantly between the treatment and control groups. In a recent study, allergic patients received a booster dose at 1 year after three pre-seasonal intralymphatic injections of birch or timothy pollen; the booster dose further decreased the symptom and medication scores during the second pollen season compared with the first pollen season [8]. However, the study was unblinded after the end of the first pollen season, and no symptom or medication scores were recorded in the control group during the second pollen season.

Witten et al. assessed the usefulness of ILAIT for treating timothy grass (*Phleum pretense*) allergy and found that seasonal symptoms, QOL, skin or nasal reactivity to allergens, intracellular cytokine levels, regulatory T-cell marker expression, and histamine release levels did not differ significantly between the treatment and control groups [11]. By contrast, Patterson et al. reported that in subjects with a timothy grass allergy, ILAIT reduced the total combined score of symptoms and medications; however, Patterson et al. used different allergen concentrations and injection intervals from those used by Witten et al. [6]. Two recent DBPC trials reported that in individuals with Japanese cedar and mountain cedar allergies, ILAIT alleviated allergic symptoms and reduced rescue medication use [9, 10]; nevertheless, further investigations are required to confirm these therapeutic outcomes. The usefulness of ILAIT for treating pollen-induced AR remains unclear [30, 31].

The clinical efficacy of ILAIT against non-pollen-induced allergies was first assessed by Senti et al. [12]. In a DBPC study, Senti et al. showed that ILAIT with recombinant cat allergens reduced nasal and skin reactivity, as well as increased cat-specific IgG4 serum levels and IL-10 production levels. In a recent open-label pilot study in China, cervical ILAIT improved nasal and ocular symptoms and QOL, as well as reduced rescue medication use in individuals with a HDM allergy [16]. However, the study did not include a control group, and the safety of cervical ILAIT was not comprehensively investigated.

Unlike the aforementioned studies, which used aluminum hydroxide-absorbed allergen extracts, in our previous open-label pilot studies, we evaluated the clinical efficacy and safety of ILAIT with aqueous allergen extracts of *D. farinae*, *D. pteronyssinus*, cat, dog, or mixtures thereof [14, 15]. Although ILAIT improved allergic symptoms and the QOL, in some study subjects, ILAIT provoked severe local and systemic hypersensitivity reactions, especially in individuals treated with aqueous HDM allergens [14, 15]. Hence, we substituted aqueous allergen extracts with tyrosine-adsorbed allergen extracts, which are considered safer [32]. However, one patient experienced a severe hypersensitivity reaction (grade 3) after ILAIT using tyrosine-adsorbed allergen extracts. With regard to local reactions, intralymphatic injection was proven to be more painful than venous puncture in this study. Previous studies also reported moderate-to-severe hypersensitivity reactions after ILAIT [5, 7]. Thus, future studies are required to improve the safety of ILAIT. Additionally, a novel AIT such as epicutaneous immunotherapy using microneedles or adhesive-tape stripping can be an alternative modality that can minimize the risks of local and systemic adverse reactions [33, 34].

The findings of this study suggest that ILAIT using tyrosine-adsorbed allergen extracts does not significantly alleviate AR induced by HDM, cat, or dog allergens. The relatively small number of subjects might have contributed to the insignificant efficacy of ILAIT for treating AR, although previous DBPC trials reported conclusive findings with a smaller number of subjects than that used in our study [2, 6, 12]. Alternatively, the ILAIT schedule (three intralymphatic injections at 4-week intervals) and doses used might have been insufficient to exert a therapeutic effect, given that most outcomes assessed did not differ significantly between the two groups. Additionally, only short-term reductions in rescue medication use, as well as partial improvements in QOL and skin reactivity response were observed after treatment.

## Conclusion

ILAIT with tyrosine-adsorbed allergen extracts had no definite therapeutic effect on AR induced by HDM, cat, or dog allergens, although it may reduce rescue medication use, and partially improve QOL and skin reactivity responses. Additionally, ILAIT may provoke a severe hypersensitivity reaction; thus, future studies are required to elucidate the clinical efficacy and safety of ILAIT with various allergen extracts.

## Supplementary Information

**Additional file 1: Fig. S1.** Mean domain score of each domain in RQLQ. **A **Emotional function. **B** Practical problems. **C** Sleep. **D** Non-nose/eye symptoms. **E** Nasal symptoms. **F** Ocular symptoms. *P < 0.05. RQLQ, rhinoconjunctivitis quality of life questionnaire.

**Additional file 2: Fig. S2.** Skin reactivity in SPT with serially diluted target allergens. **A** HDM. **B** cat. **C** dog. *P < 0.05 compared to baseline. ^#^*P* < 0.05 compared to control group. HDM allergens used in SPT consisted of both *Dermatophagoides farinae* and *D. pteronyssinus* except one subject in whom only *D. farinae* allergen was used in SPT because target allergen was *D. farinae*. SPT, skin prick test; HDM, house dust mite; MWD, mean wheal diameter.

**Additional file 3: Fig. S3.** Skin reactivity in IDT with serially diluted target allergens. **A** HDM. **B** cat. **C** dog. HDM allergens used in IDT consisted of both *Dermatophagoides farinae* and *D. pteronyssinus* except one subject in whom only *D. farinae* allergen was used in IDT because target allergen was *D. farinae*. IDT, intradermal test; HDM, house dust mite; MWD, mean wheal diameter.

**Additional file 4: Fig. S4.** Basophil reactivity represented by the percentages of CD63 + basophil activated by *Dermatophagoides farinae* (**A**), *D. pteronyssinus* (**B**), dog (**C**) and cat (**D**) allergen in basophil activation test.

## Data Availability

We will provide data and materials if requested by email (sangminlee77@naver.com).

## Abbreviations

AIT: Allergen-specific immunotherapy
AR: Allergic rhinitis
BAT: Basophil activation test
dMS: Daily medication score
dSMS: Daily symptom medication score
dSS: Daily symptom score
HDM: House dust mite
IgE: Immunoglobulin E
IDT: Intradermal test
ILAIT: Intralymphatic allergen-specific immunotherapy
NAPT: Nasal allergen provocation test
QOL: Quality of life
RQLQ: Rhinoconjunctivitis quality of life questionnaire
SNOT-20: Sinonasal outcome test-20
SPT: Skin prick test
VAS: Visual analog scale
