# Supplementary figures and images for: Intralymphatic immunotherapy with tyrosine-adsorbed allergens: a double-blind, placebo-controlled trial
Source: Respir Res. 2021 Jun 4;22:170. doi: 10.1186/s12931-021-01766-0 (PMC8178859; doi:10.1186/s12931-021-01766-0)

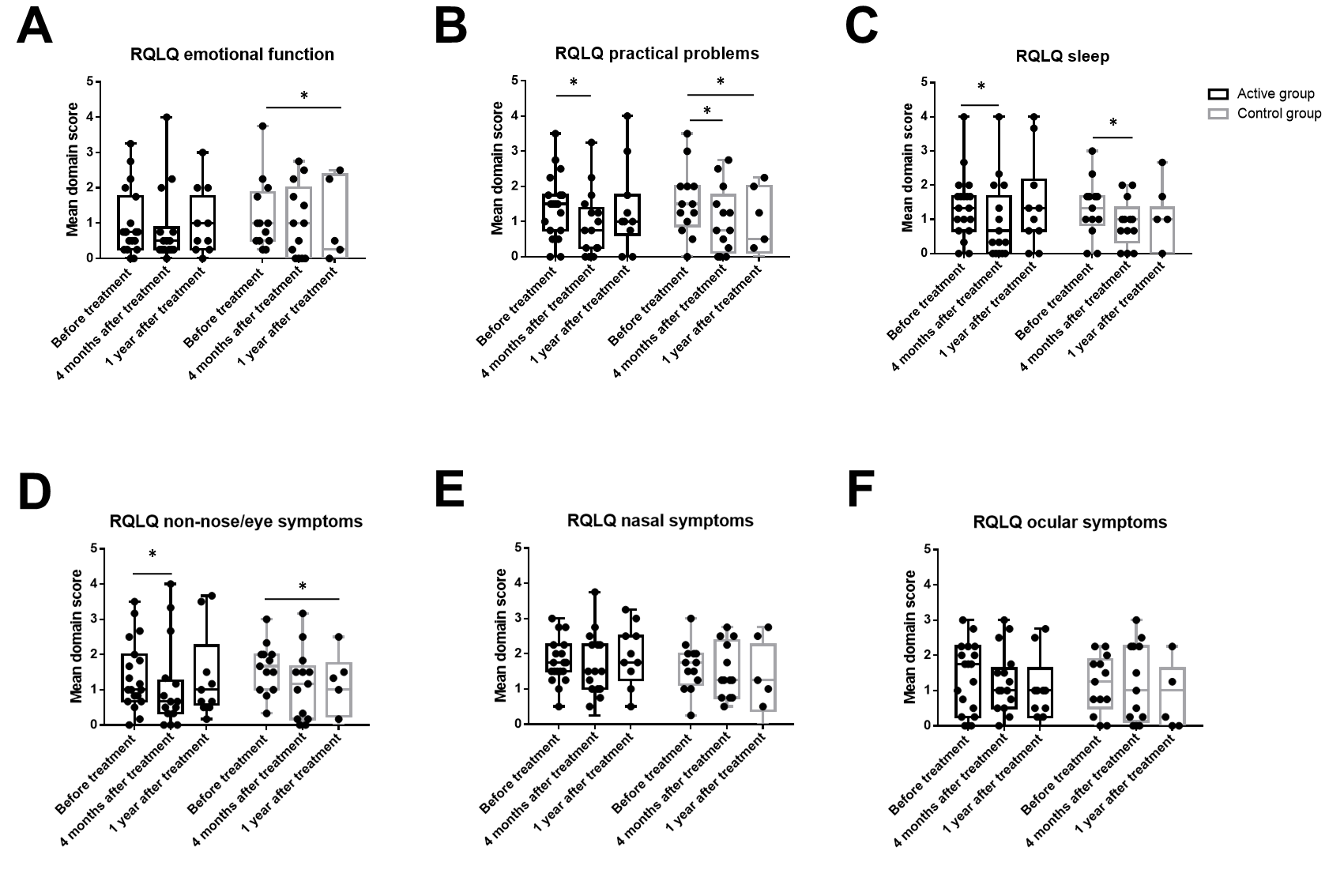

Supplement: Supplementary file 1 — Additional file 1: Fig. S1. Mean domain score of each domain in RQLQ. A Emotional function. B Practical problems. C Sleep. D Non-nose/eye symptoms. E Nasal symptoms. F Ocular symptoms. *P < 0.05. RQLQ, rhinoconjunctivitis quality of life questionnaire. [file 12931_2021_1766_MOESM1_ESM.tif]

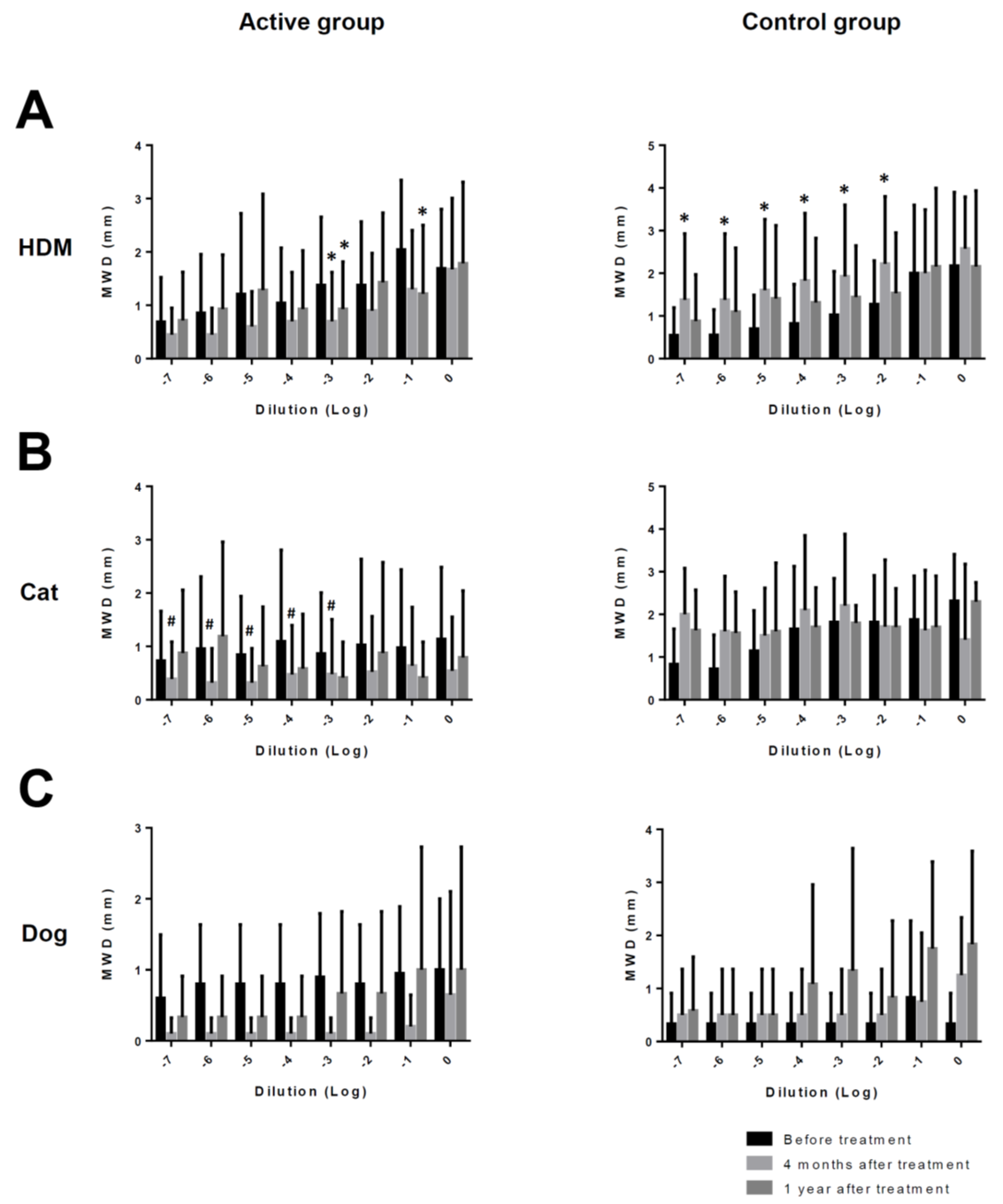

Supplement: Supplementary file 2 — Additional file 2: Fig. S2. Skin reactivity in SPT with serially diluted target allergens. A HDM. B cat. C dog. *P < 0.05 compared to baseline. #P < 0.05 compared to control group. HDM allergens used in SPT consisted of both Dermatophagoides farinae and D. pteronyssinus except one subject in whom only D. farinae allergen was used in SPT because target allergen was D. farinae. SPT, skin prick test; HDM, house dust mite; MWD, mean wheal diameter. [file 12931_2021_1766_MOESM2_ESM.tif]

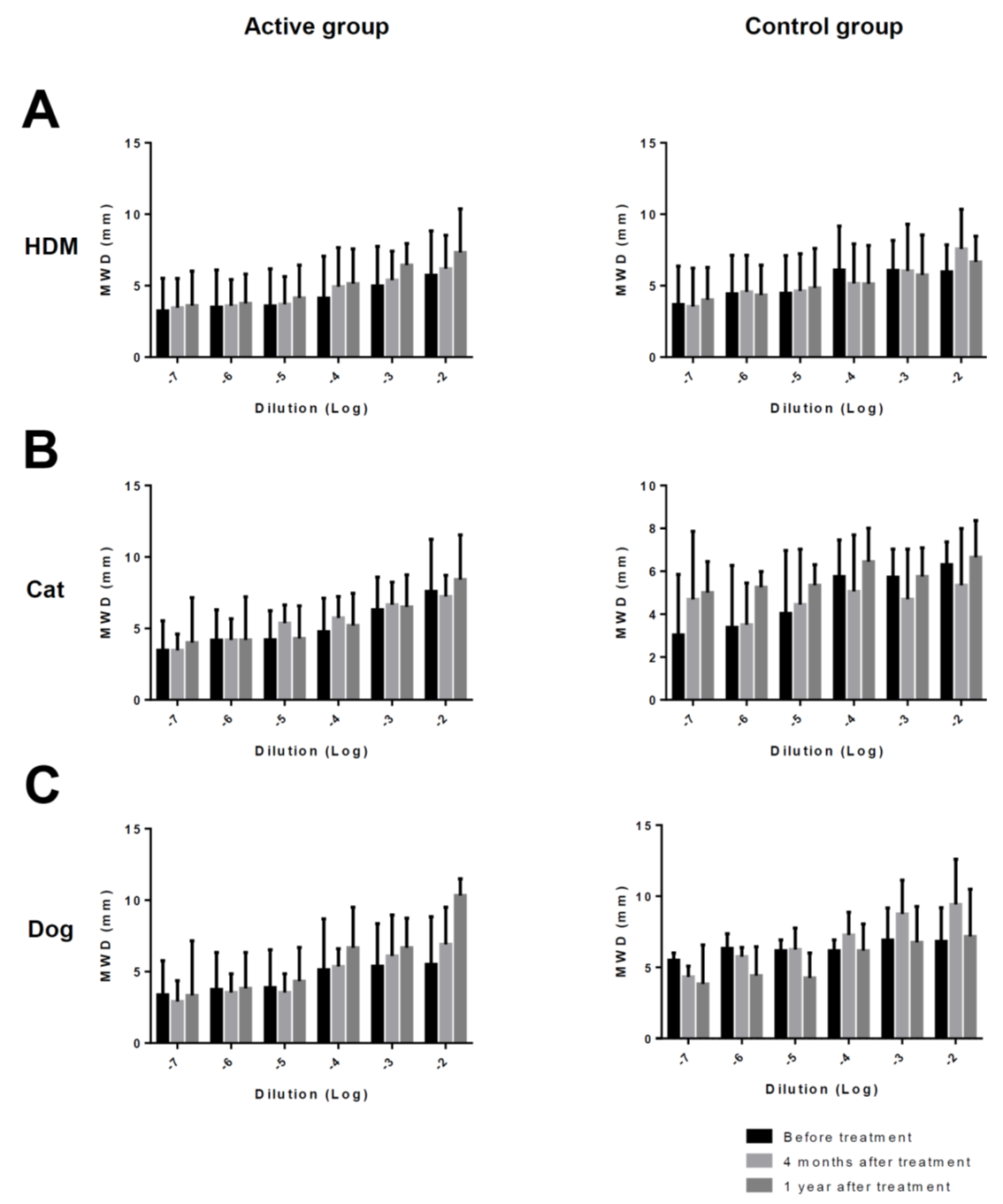

Supplement: Supplementary file 3 — Additional file 3: Fig. S3. Skin reactivity in IDT with serially diluted target allergens. A HDM. B cat. C dog. HDM allergens used in IDT consisted of both Dermatophagoides farinae and D. pteronyssinus except one subject in whom only D. farinae allergen was used in IDT because target allergen was D. farinae. IDT, intradermal test; HDM, house dust mite; MWD, mean wheal diameter. [file 12931_2021_1766_MOESM3_ESM.tif]

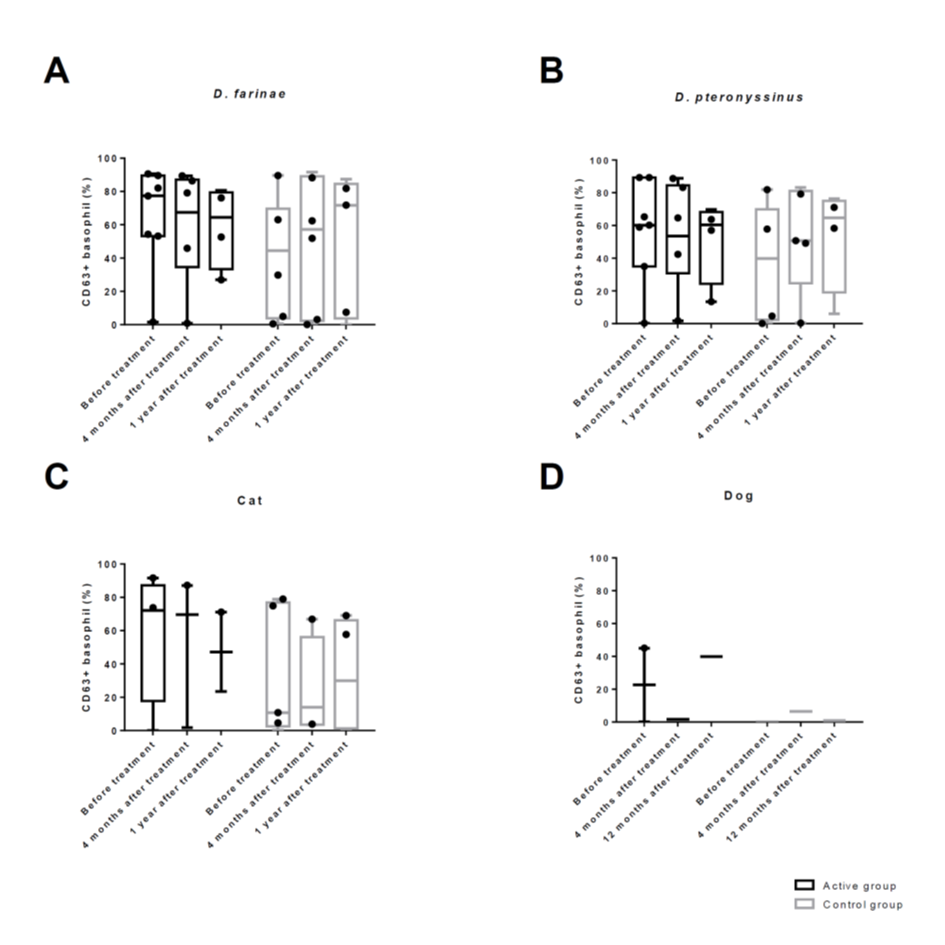

Supplement: Supplementary file 4 — Additional file 4: Fig. S4. Basophil reactivity represented by the percentages of CD63 + basophil activated by Dermatophagoides farinae (A), D. pteronyssinus (B), dog (C) and cat (D) allergen in basophil activation test. [file 12931_2021_1766_MOESM4_ESM.tif]
